# Measuring the Subjective Cost of Listening Effort Using a Discounting Task

**DOI:** 10.1044/2020_JSLHR-20-00086

**Published:** 2021-01-13

**Authors:** Drew J. McLaughlin, Todd S. Braver, Jonathan E. Peelle

**Affiliations:** a Department of Psychological & Brain Sciences, Washington University in St. Louis, MO; b Department of Otolaryngology, Washington University in St. Louis, MO

## Abstract

**Purpose:**

Objective measures of listening effort have been gaining prominence, as they provide metrics to quantify the difficulty of understanding speech under a variety of circumstances. A key challenge has been to develop paradigms that enable the complementary measurement of subjective listening effort in a quantitatively precise manner. In this study, we introduce a novel decision-making paradigm to examine age-related and individual differences in subjective effort during listening.

**Method:**

Older and younger adults were presented with spoken sentences mixed with speech-shaped noise at multiple signal-to-noise ratios (SNRs). On each trial, subjects were offered the choice between completing an easier listening trial (presented at +20 dB SNR) for a smaller monetary reward and completing a harder listening trial (presented at either +4, 0, −4, −8, or −12 dB SNR) for a greater monetary reward. By varying the amount of the reward offered for the easier option, the subjective value of performing effortful listening trials at each SNR could be assessed.

**Results:**

Older adults discounted the value of effortful listening to a greater degree than young adults, opting to accept less money in order to avoid more difficult SNRs. Additionally, older adults with poorer hearing and smaller working memory capacities were more likely to choose easier trials; however, in younger adults, no relationship with hearing or working memory was found. Self-reported measures of economic status did not affect these relationships.

**Conclusions:**

These findings suggest that subjective listening effort depends on factors including, but not necessarily limited to, hearing and working memory. Additionally, this study demonstrates that economic decision-making paradigms can be a useful approach for assessing subjective listening effort and may prove beneficial in future research.

An increasing body of research indicates that, when faced with listening challenges, such as those caused by signal degradation and hearing loss, listeners may need to recruit additional cognitive resources to support speech processing. The allocation of these cognitive resources to meet processing demands is frequently referred to as listening effort (Pichora-Fuller et al., 2016). In this context, measuring the cognitive demands a listener faces during listening has taken on an important role in advancing our understanding of the factors that contribute to successful speech comprehension and how individual differences in cognitive ability may affect communication (Peelle, 2018).

Most approaches to quantifying cognitive demands during listening have focused on objective measures of behavioral performance. For example, when speech is acoustically degraded, cognitive resources that typically support memory encoding appear to be reallocated to speech processing (i.e., due to a limited-capacity system; Kahneman, 1973; Wingfield, 2016), resulting in poorer recall. Thus, recall of what has been heard can be negatively impacted by environmental noise or signal degradation, even when recognition accuracy is fairly high (Heinrich et al., 2008; Koeritzer et al., 2018; Ward et al., 2016). Additionally, even recall of words presented in quiet can be negatively affected when intermixed in lists containing acoustically degraded words (Cousins et al., 2014; Rabbitt, 1968). Another approach to measuring cognitive demand during listening is to track physiological responses, such as the task-evoked pupil response (for a review, see Van Engen & McLaughlin, 2018). Pupils increase in diameter during task engagement, and a widely held view is that the magnitude of the pupil response reflects the amount of cognitive effort required (Beatty, 1982; Hess & Polt, 1964). Using pupillometry, it has been demonstrated that listening effort increases for young adults as signal quality worsens (Winn et al., 2015) and as the intelligibility of speech decreases (Zekveld & Kramer, 2014; Zekveld et al., 2010). Harder signal-to-noise ratios (SNRs) also elicit larger and more sustained pupil responses in older adults with hearing loss, especially when there is a lexical competitor present (Kuchinsky et al., 2013).

Complementing measures of objective cognitive demand are estimates of subjective listening effort. Subjective impressions may play an important role in seeking treatment for hearing loss and outcome assessment for assistive devices (Humes, 1999) and for understanding emotional components (such as frustration) that are not fully explained by cognitive measures (Eckert, Matthews, & Dubno, 2017). Self-report questionnaires (Gatehouse & Noble, 2004) and rating scales (Brons et al., 2014; McAuliffe et al., 2012; Picou et al., 2011) have been used to measure subjective listening effort, and many studies examining physiological indexes of effort include these measures (e.g., Zekveld et al., 2010). Other studies have used self-report and/or interviews as the primary measure. For example, in a qualitative study of 61 workers with and without hearing loss, Hétu et al. (1988) used content analysis to extract themes from unstructured interviews.

However, despite ostensibly focusing on similar aspects of effort during listening, subjective and objective measures do not always converge (Strand et al., 2018). One potential explanation for this lack of convergent validity is that self-reported subjective effort relies on metacognitive assessment; thus, while researchers may be attempting to measure a single construct of “listening effort,” participants may actually be conflating multiple factors, such as general fatigue, objective processing demand, performance, and actual expended effort (McGarrigle et al., 2014). Indeed, Moore and Picou (2018) found that subjects are influenced by their task performance when self-rating mental effort, reflecting the effect of judgment heuristics on their self-evaluations. Thus, a favorable alternative to metacognitive probes would be an experimental paradigm that reveals participant preferences in a quantitatively tractable manner.

In behavioral economics, a large class of decision-making paradigms has been developed to assess subjective value through revealed preferences, by offering rewards paired with factors such as delay or risk (Green & Myerson, 2004; Myerson & Green, 1995). For example, in delay discounting paradigms, decisions are made between larger-later and smaller-sooner options (e.g., “Would you prefer $20 today, or $80 in ten days?”; Myerson & Green, 1995). More recently, discounting paradigms have been used to examine subjective cognitive effort associated with working memory (Westbrook et al., 2013, 2019) and reading tasks (Eckert, Vaden, et al., 2017). For example, Westbrook et al. (2013) developed the cognitive effort discounting (COG-ED) task, in which participants are offered a choice on each trial between performing a harder task for more monetary reward and performing an easier task for less monetary reward, with the amount of money offered for the easier task varied across choice trials. The subjective value of performing the more difficult task was thus revealed by the amount of money that a participant is willing to “give up” to avoid expending increased effort. In the context of communication effort, Eckert, Vaden, et al. (2017) compared delay discounting (using the Monetary Choice Questionnaire; Kirby, 2009) with hearing loss and “information demand,” a measure of how many times participants opted to reglimpse visually degraded target sentences (i.e., in order to better understand them). Their results indicated that delay discounting was unrelated to hearing thresholds but was related to information demand, such that subjects who reported they would wait longer in order to receive larger rewards were also more likely to reglimpse stimuli.

The goal of assessing subjective effort in a quantitatively precise manner has long been challenging, and only recently have paradigms using objective measures been developed. To accomplish this goal, in this study we apply, for the first time, a discounting paradigm adapted from the COG-ED. Specifically, we use a speech COG-ED to examine subjective listening effort^1^ for speech in speech-shaped noise that was parametrically varied across multiple SNRs, ranging from +20 dB (very easy) to −12 dB (very difficult). We anticipated that individual differences in age, hearing, and cognitive ability would predict subjective listening cost during speech-in-noise perception. Numerous studies in the speech-in-noise perception literature have shown that listening effort and accuracy are poorer for subjects who are older (Humes, 1996; Sommers & Danielson, 1999), have smaller working memory capacities (DeCaro et al., 2016; Ward et al., 2016), and/or have greater hearing loss (Koeritzer et al., 2018; McCoy et al., 2005). Thus, we predicted that older adults would experience greater listening effort for speech-in-noise perception compared to young adults, resulting in greater “discounting” than young adults at the most difficult SNRs (i.e., accepting less monetary reward in order to avoid a harder listening task). Additionally, we tested a priori, preregistered hypotheses that measures of area under the curve (AUC) from the discounting task would positively correlate with working memory capacity and negatively correlate with hearing status; these hypotheses, in particular, were based on data indicating that subjective ratings of listening effort are related to individual differences in cognitive capacity and hearing status (Koelewijn et al., 2012; Rudner et al., 2012). Other factors, such as task performance and self-reported desire to do well, were also examined, but no hypotheses were preregistered for these analyses.

## Method

Preregistration of sample size, primary outcome measures, and exclusion criteria can be found at https://osf.io/tfc83/, and experimental materials (including experiment programs and stimuli), raw data, and analysis scripts can be found at https://osf.io/8jpnx/files/. All procedures were approved by the Washington University in St. Louis Institutional Review Board.

### Participants

Young and older adults were recruited using the Washington University Psychology Subject Pool and a local participant registry. Eligibility criteria included the following: English as a first language, no history of neurological difficulties, and no clinically diagnosed hearing loss or use of hearing aids. Five young adults were excluded and replaced due to failure to meet eligibility criteria or experiment malfunction. No older adults were excluded, but for one older adult, there was an experiment malfunction resulting in 19% trial loss; including or excluding this participant did not change the results of the analyses. The final sample included 50 young adults between the ages of 18 and 24 years (*M* = 19.9 years, *SD* = 1.5; 35 female) and 50 older adults between the ages of 65 and 79 years (*M* = 70.9 years, *SD* = 3.6; 33 female). Participants were compensated $10 per hour, $5 for travel expenses, and between $0 and $10 in bonus pay depending on choices made during the task (typically $25–$35 in total). All subjects provided informed consent prior to participation.

### Materials

Speech materials were recordings of a female native speaker of American English reading 160 sentences developed by Van Engen et al. (2012), with four key words each (e.g., “the *gray mouse ate* the *cheese*”). For the familiarization phase of the task, an additional 40 Hearing in Noise Test sentences read by another female native speaker of American English (Nilsson et al., 1994) were used as filler items to increase the length of the phase, ensuring that subjects became sufficiently familiar with the difficulty of each noise level. Sentences were presented in speech-shaped noise that was created to have the same long-term average spectrum of the sound files. Stimuli were mixed with Praat Version 6.0.16 (Boersma & Weenink, 2019) and presented at SNRs of +20, +4, 0, −4, −8, and −12 dB.

### Procedure

#### Pure Tone Audiometry

After the consent process, participants completed audiometric testing. Participants were seated in a sound-attenuating booth with headphones and a response clicker. A researcher administered the test from outside the booth. Pure tones were presented at 250, 500, 1000, 2000, 4000, and 8000 Hz. Thresholds were determined by decreasing a given tone's intensity in 10 dB intervals until the participant could not detect it and then increasing in 5 dB intervals until the participant correctly responded. The lowest intensity at which participants responded 2 or more times out of three opportunities was recorded. Thresholds at 500, 1000, and 2000 Hz were averaged to determine a pure-tone average (PTA) in each ear,^2^ and values from the better ear were used for analyses.

#### Working Memory Capacity

We used the Word Auditory Recognition and Recall Measure to measure working memory capacity (Smith et al., 2016). The Word Auditory Recognition and Recall Measure is an auditory working memory task that provides measures of word recognition and recall for sets of two to six words. Words are presented one at a time, and participants are instructed to repeat the word aloud (to confirm recognition accuracy) and judge whether the first letter of the word is from the first (A–M) or second (N–Z) half of the alphabet. After all of the words in a set have been presented, participants are cued to recall as many words from the set as possible, in order.

#### Phase 1: Familiarization

We used PsychoPy Version 1.84.1 (Peirce, 2007) for the listening experiments. To introduce the range of difficulty levels, the discounting task began with a familiarization phase. During this phase, participants were presented with sentences in noise and instructed to repeat aloud as much of each sentence as possible. An experimenter in the room typed the participant's response to minimize typing errors (particularly for older adult subjects); additionally, the presence of an experimenter provided a social incentive while performing the task. Participants were instructed to guess if they were unsure of any words in a sentence.

**Figure 1.**
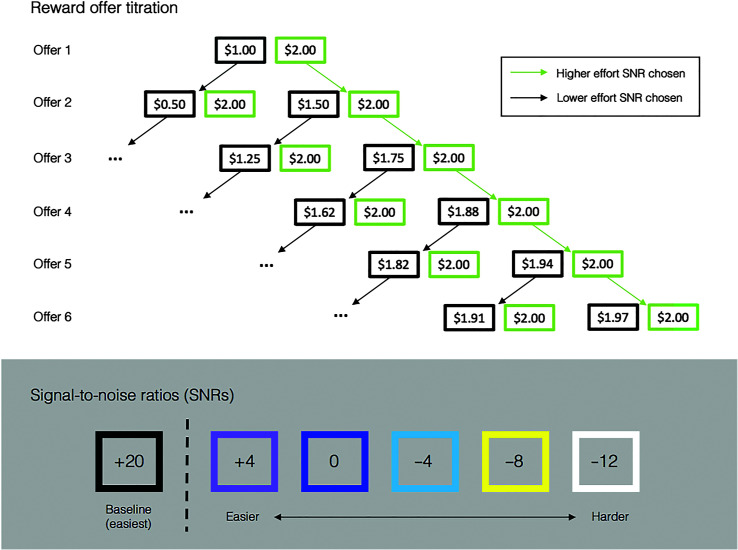
Visualization of the reward offer titration. Each block contained six trials, always beginning with Offer 1, which was a $1.00 reward for performing a black (+20 dB signal-to-noise ratio [SNR]) trial or a $2.00 reward for performing a trial at a more difficult SNR. Each subsequent offer was determined by the participant's previous choice; if the harder SNR was chosen, then the lower reward offer would increase, and if the easier SNR was chosen, then the lower reward offer would decrease. The amount of change to the lower reward offer halved in each trial. In the diagram, the higher effort condition is green, but the actual color displayed to participants corresponded to one of the five higher effort SNRs (+4, 0, −4, −8, or −12 dB; shown in the lower panel of the figure). The color of the higher effort condition changed in each block, but the titration process was the same across conditions.

There were 80 trials during the familiarization phase, 16 sentences per SNR (excluding the +20 SNR level). Half of the sentences per SNR were filler items included to increase the length of Phase 1 and are not included in the analyses. Each SNR was labeled with a color (see Figure 1) to reduce anchoring effects, which are cognitive biases that could cause subjects to base judgments off of an initial (or baseline) level of difficulty (Ariely et al., 2003). Thus, prior to the discounting phase, participants learned the difficulty of each color without an explicit numeric label. Stimuli were blocked by SNR, with four trials per block. Subjects completed blocks in order of difficulty, from easiest (i.e., 4 SNR) to hardest (i.e., −12 SNR), completing four cycles. The color of a given level was displayed on the screen prior to each trial.

#### Phase 2: Discounting

Following the familiarization phase, participants completed the discounting phase. Each trial, participants were offered the option of performing the trial at a harder SNR for a $2 reward or at an easier SNR for a lower reward amount; all offers were presented as colors (see Figure 1), and SNRs were not shown to the participant. The colors of the two difficulty levels offered in each trial (e.g., black vs. yellow, corresponding to +20 vs. −8 dB SNR) were presented on the screen with instructions to select an offer by way of key press (e.g., “Press ‘a' for $2.00” in a yellow box). Thus, because participants were extremely familiar with the difficulty of each color from the familiarization phase, there was minimal risk of uncertainty during selections. After making a selection, subjects then listened to a sentence presented at the chosen difficulty level and repeated what they heard aloud to the experimenter in the room. Prior to beginning, participants were informed that their bonus pay would be based on five of their actual choices from the task, chosen at random by the experiment. The easier trial was always +20 SNR (black), and the harder trial was either +4, 0, −4, −8, or −12 dB SNR, changing each block. The amount of reward for the easier choice was titrated to determine the lowest value that a participant was willing to accept (see Figure 1). The difference between the lowest reward selection and the maximum reward offered represents the value of performing the harder task (i.e., the amount of “discounting”). The first reward offers of a block were always $1 (for easier) and $2 (for harder), and for each subsequent trial, the amount of adjustment to the smaller offer depended on whether the participant had selected the smaller or larger offer; if the smaller offer was selected, then the next smaller offer would be decreased, and if the larger offer ($2) was selected, then the next smaller offer would be increased. The amount of adjustment halved each trial as the block progressed, as demonstrated in Figure 1. The discounting phase included 20 blocks in total, with four blocks per each of the “harder” SNRs (+4, 0, −4, −8, and −12 dB) contrasted against the “easier” SNR (20 dB). Participants completed six trials in each block, for a total of 120 trials.

Target sentences were divided into 20 lists of six using “Match” (Van Casteren & Davis, 2007). The target sentences were matched based on intelligibility scores (i.e., average proportion of key words correctly recognized) from a previous study; thus, the lists of target sentences for the discounting phase were matched for expected difficulty. Sentence lists and SNR were randomly assigned across blocks, such that all participants had a unique combination, and order, of sentence lists and SNR.

Participants were instructed that performance on the task (i.e., how many words they correctly recognized per sentence) would not affect their bonus pay. However, they were encouraged to contribute the same amount of effort during the discounting phase as they did in the familiarization phase. The advantage of ignoring accuracy (and explicitly informing participants of this fact) is that it reduces the influence on metalinguistic judgments; that is, we did not want participants to consider their potential performance *in addition to* the cognitive effort associated with the task.

#### Questionnaires

The last portion of the experiment involved a series of questionnaires. Participants first answered a survey assessing their motivation during the two phases of the discounting task. Next, they were given a demographics and an income questionnaire. Estimated annual family income was measured to control for differences in socioeconomic status (SES). Lastly, older adult participants completed the short version of the Hearing Handicap Inventory for the Elderly (Ventry & Weinstein, 1983). All participants were debriefed once the experiment was completed.

## Results

**Figure 2.**
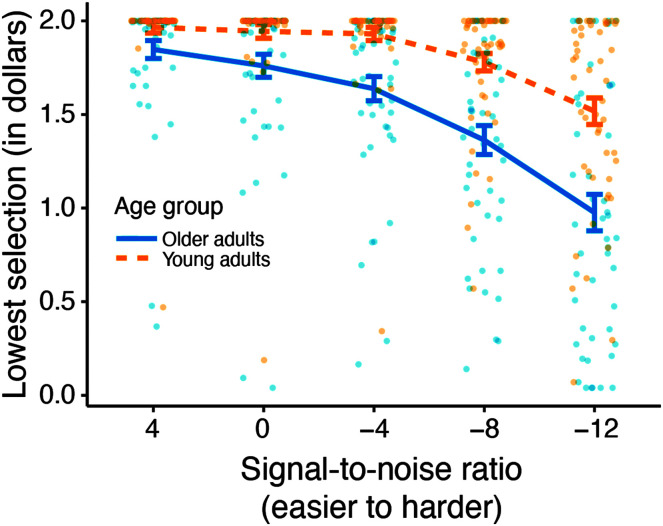
The average discounting curves are shown with 95% confidence intervals. Mean values for each participant are shown with jittered points. Lowest selection values below $2.00 indicate that the participant chose the easier (black, +20 dB) signal-to-noise ratio (SNR) in order to avoid the harder SNR listed on the *x*-axis.

Data and analysis scripts are available from https://osf.io/8jpnx/files/. Figure 2 shows the average discounting curves for young and older adults. Linear mixed-effects regression was implemented using the *lme4* package in R (Version 3.5.1) to model the discounting data. This analysis was not preregistered. However, it allowed us to control for potential intelligibility (i.e., performance) and income differences between young and older adult participants, which is crucial for interpreting the relationship between speech perception ability (i.e., working memory and hearing threshold measures) and discounting. For the model analyses, the discounting task data were summarized by block, where the value of interest was the lowest monetary amount (i.e., between $0 and $2) chosen in that block. Subjects were included as random intercepts.^3^

First, we constructed a base model with a fixed effect of SNR, which was coded as a categorical variable. The levels of SNR were ordered, such that they went in order from easiest (+4 dB) to hardest (−12 dB), where +4 dB is the reference level in the model. All levels of SNR were significant (*p*s < .05). Next, likelihood ratio tests were used to determine the significance of the effects of interest. Age Group, ordered so that the young adult group was the reference level in the model, and the Age Group × SNR interaction both significantly improved model fit (χ^2^_1_ = 18.01, *p* < .001, and χ^2^_1_ = 112.77, *p* < .001, respectively; see Figure 2). Additionally, the fixed effects of intelligibility and the Intelligibility × SNR interaction improved model fit (χ^2^_1_ = 36.66, *p* < .001, and χ^2^_1_ = 19.82, *p* < .001, respectively), as did the Intelligibility × Age Group interaction (χ^2^_1_ = 14.06, *p* < .001).^4^ Lastly, working memory capacity (χ^2^_1_ = 12.18, *p* < .001) and better ear PTA (χ^2^_1_ = 6.08, *p* = .01) were found to improve model fit, but estimated annual family income (a proxy for SES) did not (χ^2^_1_ = 0.001, *p* = .97). Thus, SES was dropped from the full model. Model estimates are reported in Table 1.

**Table 1.** Summary of the full linear mixed-effects model.

Main effect	Estimate	*SE*	*t* Value	*p*
Intercept	1.32	.26	5.15	< .001***
Block SNR (0 dB SNR)	−0.02	.20	−0.12	.91
Block SNR (−4 dB SNR)	0.42	.18	2.37	.02*
Block SNR (−8 dB SNR)	−0.04	.19	−0.21	.83
Block SNR (−12 dB SNR)	−0.35	.20	−1.74	.08
Age group (older)	−0.44	.20	−2.20	.03*
Intelligibility	0.25	.22	1.16	.25
Working memory capacity	0.09	.03	2.97	.004**
Better ear PTA	−0.01	.005	−2.45	.02*
Block SNR (0 dB SNR): age group (older)	−0.03	.05	−0.54	.59
Block SNR (−4 dB SNR): age group (older)	−0.09	.05	−1.73	.08
Block SNR (−8 dB SNR): age group (older)	0.11	.11	0.98	.33
Block SNR (−12 dB SNR): age group (older)	0.17	.16	1.07	.28
Block SNR (0 dB SNR): intelligibility	−0.003	.21	−0.02	.99
Block SNR (−4 dB SNR): intelligibility	−0.49	.19	−2.65	.008**
Block SNR (−8 dB SNR): intelligibility	−0.09	.20	−0.47	.64
Block SNR (−12 dB SNR): intelligibility	0.61	.27	2.23	.03*
Age group (older): intelligibility	0.62	.18	3.36	< .001***

*Note.* SNR = signal-to-noise ratio; PTA = pure-tone average.

* Significant at *p* < .05 level.

** Significant at *p* < .01 level.

*** Significant at *p* < .001

When controlling for all other factors, results of the full model indicated an overall effect of age group on discounting (β = −.44, *SE* = .20, *p* = .03) but no significant interactions between age group and SNR at any level (all *p*s > .05). However, preregistered direct comparisons of discounting between young and older adults were conducted at −8 and −12 dB SNR using Wilcoxon rank-sum tests and indicated that older adults made lower selections (discounted more) than younger adults (both *p*s < .001). Figure 2 demonstrates this trend with the average discounting curves for young and older adults.

The main effect of intelligibility on discounting was not significant (β = .25, *SE* = .22, *p* > .05). However, the interaction between intelligibility and age group was significant (β = .62, *SE* = .18, *p* < .001) and indicated that older adults made decisions based on their task performance to a greater degree than young adults (see Table 2). Additionally, there were significant interactions between intelligibility and SNR at −4 dB (β = −.49, *SE* = .19, *p* = .01) and −12 dB (β = .60, *SE* = .27, *p* = .03), but not at 0 or −8 dB (both *p*s > .05). These interactions reflect differences in the slope of the relationship between intelligibility and discounting at each level of SNR.^5^ Similarly, the main effect of SNR indicated that only the −4 dB level was significantly different from the reference level, +4 dB (β = .42, *SE* = .18, *p* = .02), with a marginal difference for −12 dB (β = −.35, *SE* = .20, *p* = .08) and no differences for the remaining levels (all *p*s > .05).

**Table 2.** Descriptive statistics of the familiarization phase of the discounting task, showing means with standard deviations in parentheses.

Signal-to-noise ratio	Young adults	Older adults
+4 dB	0.95 (0.05)	0.90 (0.14)
0 dB	0.97 (0.05)	0.86 (0.17)
−4 dB	0.91 (0.08)	0.66 (0.25)
−8 dB	0.56 (0.16)	0.30 (0.16)
−12 dB	0.17 (0.09)	0.07 (0.07)

*Note.* Intelligibility is a listening performance measure of the proportion of key words per sentence that the subject was able to repeat back to the experimenter.

Lastly, better ear PTA (β = −.01, *SE* = .005, *p* = .02) and working memory capacity (β = .09, *SE* = .03, *p* = .004) significantly predicted discounting, indicating subjects with poorer hearing and smaller working memory capacities tended to select lower reward values. The relationship between hearing, working memory capacity, and age group was further examined with preregistered correlation analyses. For these analyses, data from Phase 2 of the discounting task were first summarized into a single value of AUC for each subject. This value was calculated by averaging the lowest reward value a given subject selected (i.e., between $0 and $2) for each SNR, summing these lowest selection averages, and then dividing by the total possible ($10). Thus, the AUC values are all between 0 and 1, where a lower value indicates more “discounting” and a higher value indicates less “discounting.”

**Figure 3.**
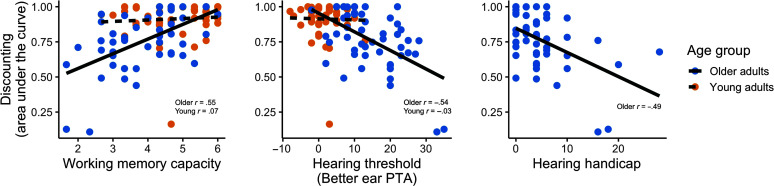
Correlations between area under the curve on the discounting task and measures of working memory capacity and hearing are shown for young and older adults. Significant correlations were found for older adults on all measures, but no significant relationships were present for young adults. Young adults did not complete the hearing handicap measure. PTA = pure-tone average (higher values correspond to poorer hearing).

Figure 3 shows correlations of discounting AUC with working memory capacity (left panel), better ear PTA (middle panel), and hearing handicap (assessed for older adults only; right panel). Pearson correlations were conducted with the *cor.test()* function in R, and Bonferroni-corrected significance level was applied for the five tests conducted (*p* < .01). For older adults, working memory capacity (*r* = .55, *p* < .001), better ear PTA (*r* = −.54, *p* < .001), and self-reported hearing handicap (*r* = −.49, *p* < .001) all significantly correlated with discounting AUC. The directions of these correlations matched our predictions, indicating that older adults who discount more tend to have smaller working memory capacities and poorer hearing. However, for young adults, neither working memory capacity (*r* = .07, *p* = .62) nor better ear PTA (*r* = −.03, *p* = .86) correlated with discounting AUC (although it is possible that this is due to a restricted range in each measure; discussed further below).

**Figure 4.**
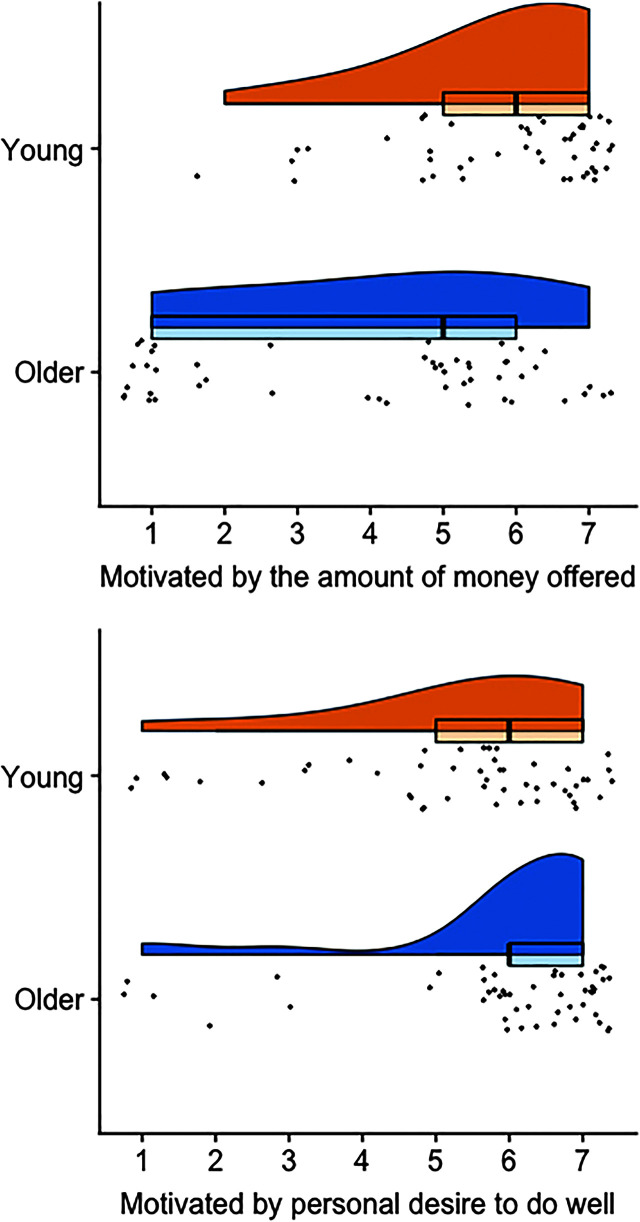
Self-reported motivation based on the amount of money offered and personal desire to do well are shown with density distributions, box plots, and jittered raw data points. Participants responded on rating scales from 1 to 7, where 1 = *not at all* and 7 = *very much*.

To further investigate what motivated participants' decisions during the task, we conducted exploratory analyses on two of the posttask survey questions: (a) “How much did the amount of money offered guide your decisions during the task?” and (b) “How much did your desire to do well guide your decisions during the task?” Participants answered on a rating scale from 1 to 7, where 1 = *not at all* and 7 = *very much*. The former question was selected because estimated income did not contribute to model fit, and the latter question was selected because of the interaction between intelligibility and age group. Figure 4 shows the distribution of data for each question and age group. Young adults reported that they were more motivated by the amount of money offered than older adults (*p* < .001), and older adults reported that they were more motivated by a desire to do well than young adults (*p* = .01), although the mean response of both groups for the latter self-report was similarly high (young adult: *M* = 5.31, older adult: *M* = 5.94). Self-reported motivation based on monetary compensation did not significantly correlate with discounting AUC for younger (*r* = .10, *p* = .51) or older (*r* = .06, *p* = .67) adults, and self-reported desire to do well predicted greater discounting (i.e., lower AUC) in young (*r* = −.38, *p* = .01), but not older (*r* = −.12, *p* = .40), adults. Thus, while young and older adults both report that they made decisions based on a desire to do well—as we expected based on the relationship between intelligibility and discounting—this relationship between discounting and reported desire to do well was only reliable for the young adults.

## Discussion

In the current study, we used a discounting paradigm (i.e., a novel speech COG-ED, adapted from the original COG-ED task; Westbrook et al., 2013) to assess subjective listening effort during speech-in-noise comprehension for young and older adult listeners. We found that older adults discounted more than young adults at difficult SNRs, settling for smaller monetary rewards to avoid expending additional cognitive effort. Differences in decision making between age groups appear to be primarily related to three factors: working memory capacity, hearing ability, and intelligibility (i.e., listening performance). In particular, older adults with poorer working memory capacities and hearing abilities discounted the most, while those with better memory and hearing behaved more similarly to young adults, who discounted less. Notably, even when controlling for all other factors, the overall effect of age on discounting remained. This indicates that there may be an additional motivational factor driving the results beyond the effects of cognitive, linguistic, and sensory abilities. Investigating what drives the difference in subjective value for listening to degraded speech in older and young adults will be a fruitful area for future research.

Results also indicated that older adults made discounting decisions based on their listening performance to a greater degree than young adults. Here, it should be reiterated that all subjects were explicitly informed that how well they performed on the listening task *would not* affect their bonus pay; for example, participants could opt to select the harder SNR for $2 every trial (guaranteeing a $10 bonus), and they would receive the full bonus regardless of how well they perceived the harder SNR stimuli. Nevertheless, it may be that older adult participants, when selecting the easier SNR option (which was always worth less reward), were choosing it in order to avoid doing poorly on task (and the negative affect associated with poor performance) and not purely to avoid additional cognitive demand. Considering that there was a researcher in the room during the task (to type the participants' verbal responses), it is also possible that older adults were more influenced by the social incentive to perform well on the task. Exploratory analyses were conducted on self-reported “desire to do well” to explore this interpretation and indicated that older adults were more motivated by a desire to do well than young adults. However, when comparing this self-report measure directly to the discounting data, a relationship only emerged for the young adult participants. Thus, it is unclear whether the interaction between intelligibility and age is related to participants' desire to do well. In future research, this could be explored by manipulating whether the researcher is in the room (i.e., manipulating the social incentive to perform well) and also by probing participants more directly regarding their affective reactions to their performance.

Individual differences in estimated annual family income (a proxy for SES) did not account for age differences in the data, but exploratory analyses of self-reported motivation did indicate that young adults' decisions, compared to older adults' decisions, were more guided by the amount of money offered. It is possible that this discrepancy is related to limitations of our income questionnaire, which did not explicitly differentiate between family- and personally earned income for the young adult group. Given that the young adult participants in this study were university students, it may be the case that they were more motivated to earn money in the task than older adults, as self-reported, but their family income did not reflect this. However, it should also be noted that, when comparing this self-report measure to the discounting data, no correlation was found for either group. Thus, it remains unclear whether older adults, in part, show greater discounting because they valued the reward offers less than the young adults. Alternatives to monetary rewards have been used in neuroeconomic delay discounting (Seaman et al., 2016) and cognitive effort (Yee et al., 2019) tasks and could be used in future listening effort research that uses discounting paradigms to compare young and older adults.

Given the novelty of the present experimental design as a measure of subjective listening cost, it should be noted that there are a number of alternate designs that can be used in future research. For example, we opted for a basic discounting design, wherein in each trial, the participants were always offered the same easy SNR (+20 dB) for a smaller monetary reward, and only the lower reward offer was titrated. This design provided some benefits for this foundational study, such as simplifying the subjective value calculation, but may also omit theoretically important contrasts. For example, the first trial in a block could provide equal monetary rewards for each offer, which might yield a stronger assessment of pure preference, and could also reveal “negative discounting” patterns, in which some participants might be willing to accept less (or equal) money to do the harder task (i.e., as could potentially occur if they had high need for cognition; Cacioppo et al., 1984). Additionally, future studies could opt to titrate the larger reward offers and/or increase the size of reward offers (e.g., $10 vs. $50); this study offered real monetary rewards, which restricted the size of rewards to a $2 maximum, but larger rewards could be offered if researchers opted for a “hypothetical” reward design (see Lagorio & Madden, 2005, for validation of this procedure). Our design also differed from the original COG-ED paradigm by requiring participants to perform a trial after every decision (as opposed to completing only a subset of repeated trials; Westbrook et al., 2013). Using a subset of repeated trials could substantially shorten the length of the experiment and allow researchers to test additional hypotheses.

For the present speech-in-noise discounting task, working memory capacity and hearing ability were related to discounting for older, but not young, adult participants. The relationships between age, working memory, hearing ability, and subjective listening cost are not surprising given the immense literature indicating that, for older adults, poorer hearing increases the difficulty of speech processing (for a review, see Mattys et al., 2012) and that working memory supports speech processing for adults with hearing impairment (Ng et al., 2013; Rudner et al., 2011); however, for young adults with normal hearing, working memory appears to play a much smaller role in supporting speech-in-noise comprehension (Füllgrabe & Rosen, 2016). However, one limitation of this study is the restricted range of the data from the hearing and working memory measures for the young adult sample. It could be the case that, in a more diverse sample of young adults with a greater range in cognitive and perceptual abilities, or when using a working memory measure with greater discriminative power (see Draheim et al., 2018), these relationships would emerge.

Neurological evidence from young adult subjects has indicated that a domain-general valuation network, including the ventromedial prefrontal cortex and ventral striatum, is engaged in decision making related to the subject costs of delay, risk, physical effort, and cognitive effort (Bartra et al., 2013; Levy & Glimcher, 2012; Westbrook et al., 2019). For subjective cognitive effort costs, Westbrook et al. (2019) also found evidence suggesting that state motivation (i.e., trial-to-trial fluctuations) may be incorporated with subjective value in ventromedial prefrontal cortex. Conversely, individual differences in cognitive effort costs were associated with activity in the ventral striatum. In future work, it will be important to determine whether the observed age differences may arise because of changes in valuation network activity among older adults or whether they are due to other neural mechanisms.

In summary, we demonstrate a novel application of a discounting task, the speech COG-ED, which relies on revealed preferences to provide a quantitative measure of subjective listening costs during speech-in-noise perception. Objective factors such as age, hearing ability, and working memory capacity are known to impact speech processing, but measuring the relationship between these factors and subjective listening effort has proven to be more difficult. Using the speech COG-ED, we were able to objectively measure subjective listening costs and determine which cognitive-, perceptual-, and motivation-based factors contributed to decision making.

## Footnotes

1 While our construct of interest is subjective listening effort, we acknowledge that the judgments in the listening task may not be solely “effort-based” judgments; rather, they likely represent an accumulation of factors that contribute to the “cost” of performance, which may include perceived difficulty, self-efficacy, motivation, and the desire to appear competent. Thus, when referring to the construct of interest, we use the term “subjective listening effort,” and when referring to how it is measured in the discounting task, we use the term “subjective listening cost.”

2 The frequencies used to calculate the PTAs are the ones typically used clinically in comparison with speech reception thresholds.

3 We were not able to include items as a random effect. The data for the mixed-effects regression analysis were not raw, by-item trial data, because the variable of interest was the lowest selection made within a block of six trials.

4 The intelligibility data used in the model are from Phase 1, not Phase 2. Intelligibility data from the concurrent task were not used because subjects did not perform an equal number of trials for each condition (and, in some cases, they could have performed no trials at a given SNR).

5 All five levels of SNR showed positive correlations with discounting. These interactions appear to be primarily driven by the restricted range of the intelligibility data, which were proportions of words correctly identified between 0 and 1. At +4 dB (the reference level), the range of the intelligibility scores is smaller (min = .34, max = 1; *M* = .93, *SD* = .11) than at −4 dB (min = 0, max = 1; *M* = .78, *SD* = .22), resulting in a shallower slope of the relationship between intelligibility and discounting. Conversely, at −12 dB, the range of intelligibility scores is smaller than that at the reference level (min = 0, max = .44; *M* = .12, *SD* = .10), resulting in a steeper slope. The 0 dB (min = .28, max = 1; *M* = .91, *SD* = .14) and −8 dB (min = 0, max = .91; *M* = .43, *SD* = .21) levels have ranges and slopes more similar to the reference level.
